# Accelerated aging in articular cartilage by ZMPSTE24 deficiency leads to osteoarthritis with impaired metabolic signaling and epigenetic regulation

**DOI:** 10.1038/s41419-023-05856-3

**Published:** 2023-05-22

**Authors:** Jinlong Suo, Rui Shao, Ruici Yang, Jinghui Wang, Zhong Zhang, Duo Wang, Ningning Niu, Xianyou Zheng, Weiguo Zou

**Affiliations:** 1 Institute of Microsurgery on Extremities and Department of Orthopedic Surgery, Shanghai Sixth People’s Hospital Affiliated to Shanghai Jiao Tong University School of Medicine, 200233 Shanghai, China; 2 State Key Laboratory of Cell Biology, CAS Center for Excellence in Molecular Cell Sciences, Shanghai Institute of Biochemistry and Cell Biology, Chinese Academy of Sciences, University of Chinese Academy of Sciences, 200031 Shanghai, China; 3 State Key Laboratory of Oncogenes and Related Genes, Stem Cell Research Center, Ren Ji Hospital, School of Medicine, Shanghai Cancer Institute, Shanghai Jiao Tong University, 200127 Shanghai, China

**Keywords:** Osteoarthritis, Senescence

## Abstract

Osteoarthritis (OA) is an age-related degenerative disease without disease-modifying therapy. The lack of aging-induced osteoarthritis models makes the discovery of therapeutic drugs more challenging. The deficiency of ZMPSTE24 could induce Hutchinson–Gilford progeria syndrome (HGPS), a genetic disorder of rapid aging. However, the relationship between HGPS and OA remains unclear. Our results found that the expression of *Zmpste24* was decreased in the articular cartilage during the aging process. *Zmpste24 knockout* mice, *Prx1-Cre; Zmpste24*^*fl/fl*^ mice and *Col2-CreERT2; Zmpste24*^*fl/fl*^ mice displayed OA phenotype. Loss of *Zmpste24* in articular cartilage could exacerbate the occurrence and development of osteoarthritis. Transcriptome sequencing revealed that deletion of *Zmpste24* or accumulation of progerin affects chondrocyte metabolism, inhibits cell proliferation and promotes cell senescence. Using this animal model, we elucidate the upregulation of H3K27me3 during chondrocyte senescence and discover the molecular mechanism by which lamin A mutant stabilizes EZH2 expression. The construction of aging-induced osteoarthritis models and the elucidation of the signaling pathways and molecular mechanisms of articular chondrocyte senescence would benefit the discovery and development of new drugs for OA.

## Introduction

Osteoarthritis (OA) is a chronic and degenerative disease in the elderly. It is mainly characterized by degenerative lesions of the articular cartilage and secondary bone hyperplasia, and is accompanied with joint swelling, pain, deformity and mobility disorders, and other disorders [1, 2]. There are still no approved disease-modifying drugs that are effective in relieving and preventing osteoarthritis [3]. The pathophysiological mechanism of osteoarthritis has not been well-understood [3, 4]. Microscopically, cartilage with OA is characterized by loss of collagen and proteoglycans [5], In the progression of OA, several degrading enzymes are produced by chondrocytes, such as Mmp13 (Matrix metalloproteinase 13) and Adamts-5 (A disintegrin and metalloproteinases with thrombospondin motifs 5) [5–7]. Chondrocytes act as the only cell group in cartilage tissue, osteoarthritis will disrupt the balance of anabolic and catabolic metabolism in chondrocytes [4, 8]. Therefore, studying the balance mechanism of chondrocyte homeostasis is helpful for drug development of OA.

The accumulation of senescent cells has been confirmed to play an important role in the occurrence and development of OA [8]. Clearing senescent cells in articular cartilage and synovium can effectively relieve OA [9]. However, the contribution of senescent cells to disease progression is not fully understood. The accumulation of senescent cells in articular cartilage and synovium was also seen in common OA animal models, including DMM (Destabilization of the Medial Meniscus) model and ACLT (Anterior Cruciate Ligament Transection) model [9, 10]. In terms of transgenic animal models, previous studies have generated several OA-related animal models by disrupting couple of signaling pathways, such as EGFR [11], PPARγ [12], β-catenin [13], etc. Transgenic animal models of osteoarthritis that mimic aging are still lacking. The lack of aging-induced osteoarthritis models makes the discovery of therapeutic drugs more challenging.

ZMPSTE24 is a metalloprotease required for the mature of lamin A, which maintains the structural integrity of the nucleus [14–17]. Lamin A is synthesized firstly as a precursor, prelamin A, which terminates in the C-terminal CAAX motif [17], and prelamin A undergoes four sequential post-translational modifications including isoprenylation of cysteine with a farnesyl lipid moiety, endoproteolytic removal of the -AAX peptide by ZMPSTE24, and carboxyl methylation [18]. Unlike most other CAAX proteins, prelamin A needs to undergo the second cleavage event to yield mature lamin A, which is also mediated by ZMPSTE24 [18]. Loss of *Zmpste24* results in the accumulation of partially processed prelamin A in mouse embryonic fibroblasts (MEFs), and *Zmpste24*-null mice display many premature aging features of HGPS patients [19]. One of the remarkable features of HGPS dermal fibroblasts and cells derived from *Zmpste24*^*–/–*^ embryos is the abnormal nuclear shape [20–23]. In addition, nuclear architecture abnormalities are also accompanied by the natural-aging process in the cells of clinical patients [24]. However, patients with progeria, which is caused by progerin (the abnormal of lamin A), are rarely described to harbor osteoarthritis. Moreover, the role of progeria-causing genes in osteoarthritis is unclear.

In this study, we demonstrated that the expression of ZMPSTE24 is gradually decreased during natural aging, and *Zmpste24* deficiency inhibited cell proliferation and exacerbated aging of chondrocyte. Further studies exhibited that loss of *Zmpste24* in mesenchymal stem cells and chondrocytes brought about severe osteoarthritis. The establishment of this animal model would shorten the period to study aging-related osteoarthritis, and provide a therapeutic target for the treatment of osteoarthritis. Transcriptome sequencing revealed that deletion of *Zmpste24* or accumulation of progerin affects chondrocyte metabolism, inhibits cell proliferation, and promotes cell aging. Moreover, H3K27me3 epigenetic regulation was changed in this accelerated aging osteoarthritis model.

## Materials and methods

### Mouse lines

*Zmpste24 ko*-*first* mice (*Zmpste24*^*-/-*^) and *Zmpste24*^*fl/fl*^ mice were obtained from the CAM-SU Genomic Resource Center (Clone No: EPD0705_3_A07). *Zmpste24*^*fl/fl*^ mice were crossed with the *Prx1-Cre* (a gift from Andrew McMahon, Harvard University) and *Col2-CreERT2* (a gift from professor Di Chen, Research Center for Human Tissues and Organs Degeneration, Shenzhen Institute of Advanced Technology, Chinese Academy of Sciences, Shenzhen, China) strain to generate *Prx1-Cre;Zmpste24*^*fl/fl*^ and *Col2-CreERT2; Zmpste24*^*fl/fl*^ mice. All mice analyzed were maintained on the C57BL/6 background. All mice were monitored in a specific pathogen–free (SPF) environment and treated in strict accordance with protocols approved by the Shanghai Institute of Biochemistry and Cell Biology, Shanghai Institutes for Biological Sciences, Chinese Academy of Sciences.

### RNA sequencing and raw data storage

We extracted articular chondrocytes from *Zmpste24*^*f/f*^ mice and infected them with GFP/Cre lentivirus, and obtained RNA from both Control (GFP-treated group) and CKO (Cre-treated group) groups of cells for transcriptome sequencing (fold change greater than 1.5 and *P* value less than 0.05). The data were analyzed on the free online platform of Majorbio Cloud Platform (www.majorbio.com). The raw sequencing data is stored on the Figshare page (10.6084/m9.figshare.22558324). We overexpressed progerin in the C3H10 cell line and performed RNA-sequencing analysis (fold change greater than 1.5 and *P* value less than 0.05). The raw sequencing data is stored on the Figshare page (10.6084/m9.figshare.22558282).

### Antibodies

Anti-Flag (F-3165, 1:5000, Sigma), anti-HA (SC-7392, 1:2000, Santa Cruz Biotechnology), anti-MYC (AE010, 1:1000, Abclonal Technology), Anti-TUBULIN (SC-23948, 1:10000, Santa Cruz Biotechnology). Anti-Aggrecan (13880-1-AP,1:1000, Proteintech); Anti-MMP13 (18165-1-AP, 1:1000, Abcam); Anti-ADAMTS5 (PA5-14350, 1:1000, Thermo); Anti-ZMPSTE24(A-8858, IHC 1:50, ABclonal). Anti-H3K27me1 (2500674, 1:1000, Millipore), Anti-H3K27me2 (ab24684, 1:1000, Abcam), Anti-H3K27me3 (07-449, 1:1000, Millipore), anti-EZH2 (5246, 1:1000, CST), anti-Suz12 (sc-271325, 1:1000, Santa Cruz), anti-EED (2514035, 1:1000, Millipore).

### Cell culture

HEK-293T cells were maintained in DMEM (Corning) medium supplemented with 10% fetal bovine serum (FBS) and 1% penicillin/streptomycin (PS, Gibco) solution. ATDC5 cells were maintained in DMEM/F12 (1:1) (Corning) medium supplemented with 5% FBS and 1% PS solution. Primary chondrocyte progenitor cells were obtained from the articular cartilage of neonatal mice and cultured in α-MEM medium (Corning) supplemented with 10% FBS and 1% PS solution. All cells were cultured at 37 °C in a humidified incubator with 5% CO_2_. It has been confirmed before use that there is no contamination by bacteria, mycoplasma, etc.

### Isolation of mouse primary chondrocyte progenitor cells

We collected chondrocyte progenitor cells from the condyle cartilage of 0–4-day-old mice. Condyle cartilage was first digested in digestion buffer [50 ml α-MEM containing 10% fetal bovine serum, 2 mM l-glutamine (Corning), 1 mM sodium pyruvate (Corning), 10 mM HEPES buffer (Corning), 1% MEM nonessential amino acids (Corning) and 1% penicillin/streptomycin (Gibco), 50 mg collagenase (Sigma), 100 mg Dispasea (Roche)] for 1 h under 37 °C, discard the digestion and then digested in the half concentration of digestion buffer overnight. The next day, digestion was filtered through a 70-micron cell filter and cultured in α-MEM supplemented with 10% fetal bovine serum and 1% penicillin/streptomycin (Gibco) solution. Mouse primary chondrocyte progenitor cells in passage one were used in this study.

### Micromass culture and alcian blue staining

The micromass culture was performed when chondrocyte progenitor cells reached 80–90% in a 10-cm dish or six-well plate. Cells were digested and suspended to 1 × 10^7^ cells/mL. A droplet of 12.5 μL cell suspension was plated into the center of a 24-well plate, stood at 37 °C for 2 h, and then add 500 μL chondrogenic differentiation medium (DMEM containing 10 ng/mL TGFβ3 (PeproTech, AF100-36E), 100 nM dexamethasone (Sigma), 50 μg/mL l-ascorbic acid 2-phosphate (Sigma, A8960), 1 mM sodium pyruvate (Sigma, 25-000-CIR), 40 μg/mL proline (Sigma, P5607) and 1% ITS (Cyagen, ITSS-10201-10). Micromass was acidified with 0.1 N HCl and then stained with 1% alcian blue (Sigma) on the 7th day.

### Real-time RT-PCR analysis

Total RNA was isolated from cells with TRIzol reagent (T9424, Sigma) and first-strand cDNA was synthesized from 0.5 μg total RNA using the PrimeScript™ RT Reagent Kit (PR037A, TakaRa). The real-time reverse transcriptase RT-PCR reaction was performed with the BioRad CFX96 system. Gene expression analysis from RT-PCR was quantified relative to *Hprt*.

### Radiographic assessment

#### For X-ray analysis

All of the mice were euthanized by carbon dioxide, and the hindlimbs or whole bodies were kept in 70% ethanol. The skeletons were analyzed using Faxitron MX-20 for X-ray image analysis.

#### For μ-CT analysis

Preparation of skeletal tissue and μ-CT analysis were performed as previously described [25]. The mouse femurs isolated from age- and sex-matched mice were skinned and fixed in 70% ethanol. Scanning was performed with the instrument μ-CT system SkyScan1176 (Bruker Biospin). The mouse femurs were scanned at a 9-m resolution for quantitative analysis, and the mouse knee joint were scanned at a 25-μm resolution for qualitative analysis. 3D images were reconstructed using a fixed threshold.

### Histology and immunofluorescence

Hematoxylin–eosin stain and immunohistochemistry were performed as previously described [26]. Tissue sections were used for safranin O staining according to the standard protocol. Tissues were fixed in 4% paraformaldehyde for 48 h and incubated in 15% DEPC-EDTA (pH 7.8) for decalcification. Then, specimens were embedded in paraffin and sectioned at 7 μm. Immunofluorescence was performed as previously described [27]. Sections were blocked in PBS with 10% horse serum and 0.1% Triton for 1 h and then stained overnight with anti-PCNA antibody (SC-56). Donkey-anti-rabbit Alexa Fluor 488 (1:1000; Molecular Probes, A21206) was used as secondary antibodies. DAPI (Sigma, D8417) was used for counterstaining. Slides were mounted with anti-fluorescence mounting medium (Dako, S3023), and images were acquired with a Leica SP5 and SP8 confocal microscope. DIG labeled in situ hybridization (Roche) and immunohistochemical staining (Dako). OA Research Society International (OARSI) histopathological scores follow the literature that has been reported [28].

### SA-β-gal staining

SA-β-gal staining was performed using Senescence β-Galactosidase Staining Kit (CST,9860 s) according to the Cell Signaling Technology protocol [29].

### Cell proliferation assay

Cell proliferation assays were performed according to the procedure provided in the Cell Couniting Kit 8 (Sangon Biotech, Shanghai, China).

### Study approval

All experiments were performed according to the protocol approved by the Animal Care and Use Committee of the Institute of Biochemistry and Cell Biology, SIBS, CAS.

## Results

### The expression of ZMPSTE24 is decreased during natural aging

**Fig. 1:**
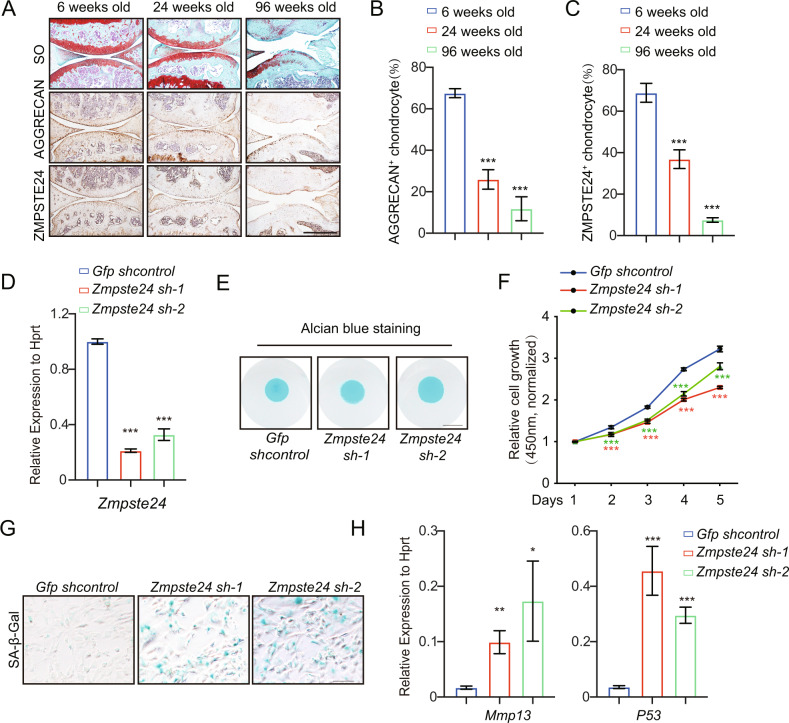
ZMPSTE24 deficiency in chondrocytes inhibits proliferation and accelerates senescence. **A** Representative images showing Safranin O and Fast Green (SO) staining of articular cartilage from mouse joints of different ages (Top). Immunohistochemistry analysis of AGGRECAN (Middle) and ZMPSTE24 (Bottom) expression in wild-type mice during aging. Scale bar = 500 μm. **B** Statistical analysis of the percentage of AGGRECAN^+^ chondrocytes in articular cartilage from (**A**). Data represent the mean ± SD, ***P < 0.001, with one-way ANOVA followed by Turkey’s test. **C** Statistical analysis of the percentage of ZMPSTE24^+^ chondrocytes in articular cartilage from (**A**). Data represent the mean ± SD, ***P < 0.001, with one-way ANOVA followed by Turkey’s test. **D** Gene expression of *Zmpste24* was detected after the primary chondrogenic progenitor cells were infected with *Gfp shRNA* (*Gfp shcontrol*) and *Zmpste24 shRNAs* (*Zmpste24sh-1*, *Zmpste24sh-2*) lentivirus. The RNA levels normalized with HPRT were shown as fold change relative to control. Data represent the mean ± SD, ***P < 0.001, with one-way ANOVA followed by Turkey’s test. **E** Primary chondrocyte progenitor cells were induced to differentiate after infection with shRNA lentivirus and stained with alcian blue (*n* = 3; three independent experiments). Scale bar=5 mm. **F** The proliferation rates of primary chondrocyte progenitor cells infected with Zmpste24 shRNAs were detected by cell proliferation assay. Absorbance was measured at 450 nm wavelength. *n* = 5 per group. **G** SA-β-gal staining of the primary chondrocyte progenitor cells were infected with Gfp shRNA and Zmpste24 shRNAs lentivirus at passage 3. Scale bar = 100 μm. **H** Genes expression of *Mmp13* and *P53* is detected in (**H**) assay. Data represent the mean ± SD, ***P < 0.001, **P < 0.01, *P < 0.05, with one-way ANOVA followed by Turkey’s test.

In order to establish an animal model of chondrocyte senescence, we found that with the increase of age, the articular cartilage gradually became degenerated and displayed osteoarthritis features with the decreased expression of chondrocyte anabolic marker AGGRECAN. Interestingly, the expression of ZMPSTE24 also decreased significantly (Fig. 1A–C). The decrease of HGPS causative gene *Zmpste24* during physiological aging prompted us to further examine the role of *Zmpste24* in cartilage homeostasis and chondrocyte senescence.

### ZMPSTE24 deficiency in chondrocytes exhibit accelerated senescence

To investigate the potential roles of ZMPSTE24 in chondrocyte, we first silenced *Zmpste24* gene with two *short hairpin RNAs* (*shRNAs*) in mouse primary chondrocyte progenitor cells (Fig. 1D) and found that knockdown of *Zmpste24* slightly inhibited chondrogenic differentiation (Fig. 1E). In addition, *Zmpste24* knockdown affected the growth rate of primary chondrocyte progenitor cells as determined by CCK-8 method (Fig. 1F). However, overexpression of *Zmpste24* gene in primary chondrocyte progenitor cells did not affect the differentiation and proliferation of cells into chondrocytes. (Supplementary Fig. 1A–C). This may be related to the function of basal level of *Zmpste24* expression in young cells. Increased levels of senescence-associated–β-galactosidase (SA-β-gal) activity and increased expression of *Mmp13* and *P53* were detected in *Zmpste24*-knockdown primary chondrocyte progenitor cells (Fig. 1G, H). These results are consistent with previous reports that the deletion of *Zmpste24* accelerates cellular senescence in chondrocytes. Taken together, these data suggest that ZMPSTE24 protect chondrocytes from premature senescence.

### ZMPSTE24 deficiency cause OA in mice

**Fig. 2:**
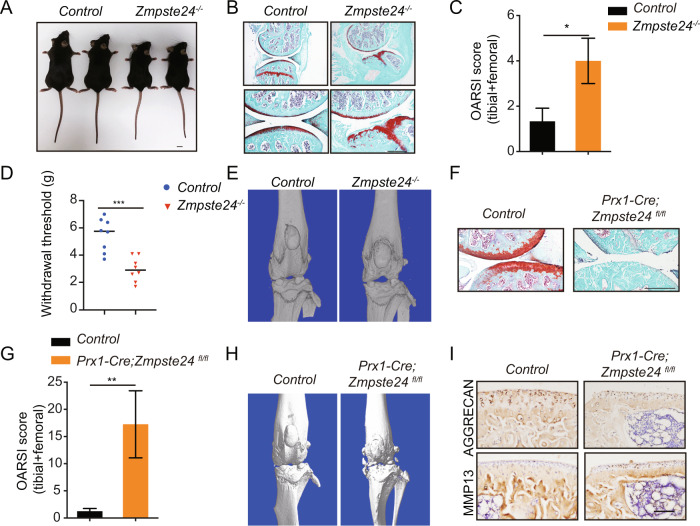
ZMPSTE24 deficiency cause OA in mice. **A** Representative images of 16-week-old *Zmpste24*^−*/−*^ mice and *Control* littermates. Scale bar = 1 cm. **B** Representative images of SO staining of hindlimbs from 16-week-old *Zmpste24*^−*/−*^ mice and *Control* littermates (*Control*: *n* = 4, *Zmpste*^*−/−*^: *n* = 3). Scale bar = 200 μm. **C** OARSI scores of articular joints (*Control* and *Zmpste24*^−*/*−^ mice). Data are presented as the mean ± SD, *P < 0.05, with unpaired Student *t* test (*Control*: *n* = 4, *Zmpste*^*−/−*^: *n* = 3). **D** Reduced sensitivity to mechanical stimulation was observed in 16-week-old *Zmpste24*^*−/*−^ mice compared to *Control* mice. Data are presented as the mean ± SD, ***P < 0.001, with unpaired Student *t* test. (*n* = 4 per group, 2 replicates per sample). **E** Representative reconstructed μ-CT images of the tibial plateau from *Control* and *Zmpste24*^*−/*−^ mice at 16-week-old (*n* = 3 per group). **F** Representative images of SO staining of hindlimbs from 25-week-old *Prx1-Cre;Zmpste24*^*fl/fl*^ mice and *Control* littermates (*n* = 4 per group). Scale bar = 200 μm. **G** OARSI scores of articular joints (*Control* and *Prx1-Cre;Zmpste24*^*fl/fl*^ mice). Data are presented as the mean ± SD, **P < 0.01, with unpaired Student *t* test (*n* = 4 per group). **H** Representative reconstructed μ-CT images of the tibial plateau from *Control* and *Prx1-Cre;Zmpste24*^*fl/fl*^ mice at 25-weeks-old. **I** Representative images of immunohistochemical staining of AGGTECAN and MMP13 in sagittal sections of 25-week-old *Control* and *Zmpste24*^−*/−*^ mice knee joints. Scale bar = 100 μm.

To determine the contribution of *Zmpste24* to the occurrence and development of osteoarthritis in vivo, we constructed *Zmpste24* transgenic knockout mice. We first analyzed that *Zmpste24 ko-first* mice (*Zmpste24*^−*/*−^*)* achieved knockout of ZMPSTE24 in articular cartilage cells and found that the knockout mice had joint stiffness and abnormal articular cartilage wear (supplementary Fig. 1d and Fig. 2A, B). OA Research Society International (OARSI) histopathological scores showed that *Zmpste24* deletion exacerbated the development of osteoarthritis (Fig. 2C). Loss of *Zmpste24* also exacerbated joint pain in *Zmpste24*^−*/−*^ mice (Fig. 2D). Although we did not see significant osteophyte formation in our μ-CT results, we found that loss of *Zmpste24* resulted in abnormal joints with rough joint surfaces (Fig. 2E). Next, we crossed *Prx1-Cre* tool mice with *Zmpste24*^*fl/fl*^ mice to construct an animal model for knocking out *Zmpste24* in mesenchymal stem cells. The 25-week-old *Prx1-Cre; Zmpste24*^*fl/fl*^ mice developed severe osteoarthritis with articular cartilage wear, subchondral bone hyperplasia, reduced proteoglycan staining, and a marked increase in periarticular osteophytes (Fig. 2F–H). In addition, immunohistochemical analysis demonstrated that the expression of chondrocyte anabolic marker AGGRECAN was decreased. We also found that the expression of chondrocyte catabolic marker MMP13 was increased (Fig. 2I). These results suggest that the deletion of *Zmpste24* induces osteoarthritis with accelerated aging.

### ZMPSTE24 deficiency in articular chondrocytes cause OA in mice

To further elucidate the role of *Zmpste24* in articular chondrocytes and determine the contribution of articular chondrocyte senescence to OA development, we employed a conditional gene knockout mouse in which the *Zmpste24* gene was specifically inactivated in chondrocytes (*Col2-CreERT2; Zmpste24*^*fl/fl*^) [30, 31]. *Col2-Cre* activity was confirmed by the expression of Tdtomato in *Col2-Cre; ROSA26-Ai9* reporter mice. And 2-week-old *Col2-CreERT2; Zmpste24*^*fl/+*^ mice were intraperitoneally injected with tamoxifen 3 times a week (Supplementary Fig. 2a). Tdtomato activity was detected in articular chondrocytes, growth plate and epiphyseal bone, but little activity was observed in the synovium and ligament (Supplementary Fig. 2b).

**Fig. 3:**
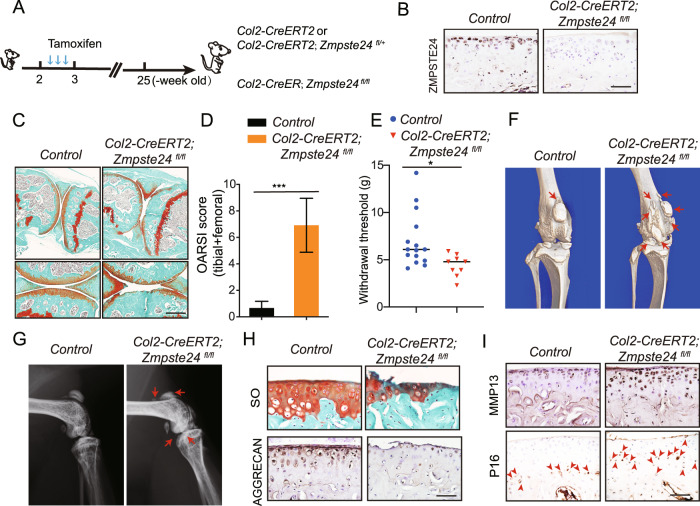
ZMPSTE24 deficiency in articular chonrocytes cause OA in mice. **A** Schematic diagram showing the protocol of tamoxifen administration for ablating ZMPSTE24 in articular chondrocytes. Three successive doses of tamoxifen were injected in a week since 2-week-old. Knee joints were analyzed at 25-week-old. **B** Representative images of immunohistochemical staining of ZMPSTE24 in 25-week-oldarticular cartilage from *Col2-CreERT2; Zmpste24*^*fl/fl*^ mice and *Control* mice. Scale bar = 100 μm. **C** Representative images of SO staining of hindlimbs from 25-week-old *Col2-CreERT2; Zmpste24*^*fl/fl*^ mice and *Control* littermates (*n* = 6 per group). Tamoxifen was injected since 2-week-old. Scale bar = 200 μm. **D** OARSI scores of articular joints (*Control* and *Col2-CreERT2; Zmpste24*^*fl/fl*^ mice). Data are presented as the mean ± SD, ***P < 0.001, with unpaired Student *t* test (*n* = 6 per group). **E** Reduced sensitivity to mechanical stimulation was observed in 25-week-old *Col2-CreERT2; Zmpste24*^*fl/fl*^ mice compared to *Control* mice. (Experimental group: *n* = 3; Control group: *n* = 5, 3 replicates per sample) Data are presented as the mean ± SD, *P < *0.05*, with unpaired Student *t* test. **F** Representative reconstructed μ-CT images of the tibial plateau from *Control* and *Col2-CreERT2; Zmpste24*^*fl/fl*^ mice at 25-week-old (*n* = 3 per group). **G** Representative radiographic images of the tibial plateau from *Control* and *Col2-CreERT2; Zmpste24*^*fl/fl*^ mice at 25-week-old (*n* = 3 per group). **H** Representative images of immunohistochemical staining of AGGTECAN in sagittal sections of 25-week-old *Control* and *Col2-CreERT2; Zmpste24*^*fl/fl*^ mice knee joints. Scale bar = 100 μm. **I** Representative images of immunohistochemical staining of MMP13 and P16 in sagittal sections of 25-week-old *Control* and *Col2-CreERT2; Zmpste24*^*fl/fl*^ mice knee joints. Scale bar = 100 μm.

To investigate the function of ZMPSTE24 in OA pathogenesis, 2-week-old *Col2-CreERT2; Zmpste24*^*fl/fl*^ mice and *Control* littermates were intraperitoneally injected with tamoxifen 3 times a week, and histology analysis was performed on the knee joints of 25-week-old mice (Fig. 3A). After the induction of Tamoxifen, the size of the mice did not change significantly until 25 weeks when the mice were harvested. The knockout efficiency was examined by immune-histochemical staining detection of ZMPSTE24 in knee joint cartilage (Fig. 3B). The histological severity of experimental OA was demonstrated by SO staining (Fig. 3C). From the tissue staining results, we found that the subchondral bone was thickened and articular cartilage was worn severely. OARSI histopathologic scores were significantly higher for the *Zmpste24*-deleted mice compared with *Control* mice (Fig. 3D). Loss of *Zmpste24* also exacerbated joint pain in *Col2-CreERT2; Zmpste24*^*fl/fl*^ mice (Fig. 3E). μ-CT and radiographic analysis results also demonstrated the lesions in *Col2-CreERT2; Zmpste24*^*fl/fl*^ mice which showed subchondral bone thickening and osteophytes formation (Fig. 3F, G).

In addition, immunohistochemistry analysis demonstrated that expression of chondrocyte anabolic marker AGGRECAN (ACAN) was decreased in *Col2-CreERT2; Zmpste24*^*fl/fl*^ mice at 25-week-old after tamoxifen induced (Fig. 3H), and the expression of chondrocyte catabolic metabolism markers MMP13 was increased (Fig. 3I, top). In addition, the expression of aging maker P16 was increased in *Col2-CreERT2; Zmpste24*^*fl/fl*^ mice (Fig. 3I, bottom). Thus, these results suggest that loss of *Zmpste24* in postnatal articular chondrocytes accelerates chondrocyte senescence and promotes the development of OA by inhibiting chondrocyte anabolism and activating chondrocyte catabolism.

### Absence of Zmpste24 in chondrocytes inhibits chondrocyte metabolism and proliferation

**Fig. 4:**
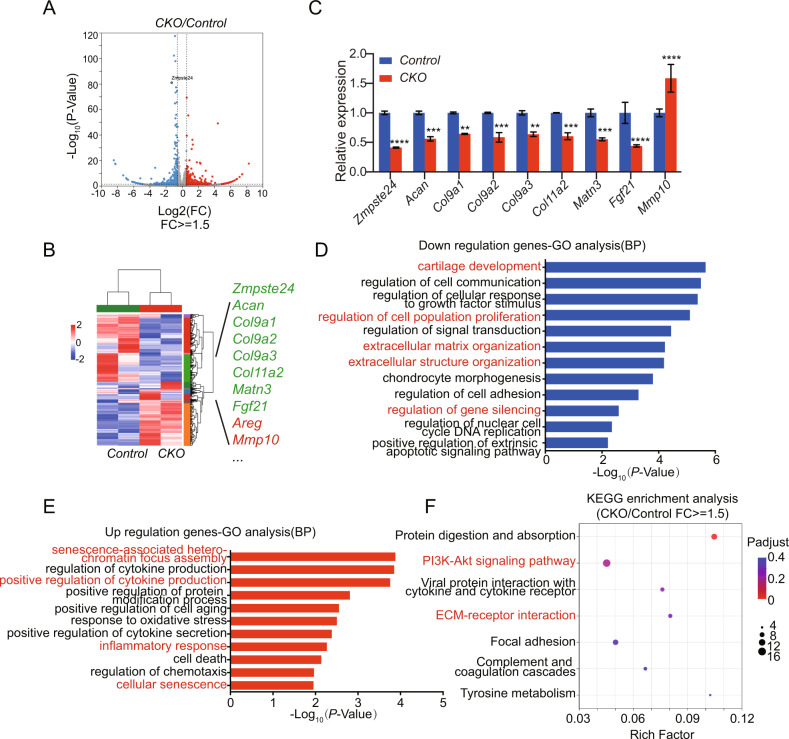
Deficiency of Zmpste24 in chondrocytes inhibits chondrocyte metabolism and proliferation. **A** Volcano plot showing the number of differential genes between the CKO group and the control group. **B** Heatmap of RNA-sequencing data between CKO and control groups. Green represents downregulated genes, red represents upregulated genes. **C** Gene expression of genes related to cartilage development and extracellular matrix organization that varied significantly in RNA-sequencing results. Data represent the mean ± SD, **P < 0.005, ***P < 0.001, ****P < 0.0001, with two-way ANOVA analysis. **D** GO analysis (Biological Pathway) of significantly downregulated (blue) genes in CKO group versus control group. **E** GO analysis (Biological Pathway) of significantly upregulated (red) genes in CKO group versus control group. **F** Signal pathway analysis of genes significantly in CKO group compared with control group.

We next explored the molecular mechanism of *Zmpste24* regulating chondrocyte aging and mature. We extracted articular chondrocytes from *Zmpste24*^*fl/fl*^ mice and infected them with GFP/Cre lentivirus, and obtained RNA from both *Control* (GFP-treated group) and *CKO* (Cre-treated group) groups of cells for transcriptome sequencing. RNA-sequencing results showed a significant reduction in *Zmpste24* expression in the *CKO* group (Fig. 4A). The heatmap also showed that marker genes related to chondrocyte anabolism, such as *Acan*, *Col9a1*, *Col9a2*, *Col9a3* etc., were also significantly downregulated (Fig. 4B, C). These results confirm that *Zmpste24* is an associated gene of natural aging. The biological functions of downregulated genes were mainly related to cartilage development, regulation of cell population proliferation, extracellular matrix organization, extracellular structure organization, regulation of gene silencing (Fig. 4D). The biological functions of upregulated gene groups are mainly related to inflammatory response and cellular senescence (Fig. 4E). Signaling pathway analysis suggest that *Zmpste24* deletion changed the expression of different genes which are mainly related to the PI3K-Akt signaling pathway and Focal adhesion (Fig. 4F). Taken together, the deficiency of *Zmpste24* in chondrocytes inhibits chondrocyte metabolism and proliferation, and enhances chondrocyte senescence.

### Progerin inhibits cell proliferation and promotes inflammation

**Fig. 5:**
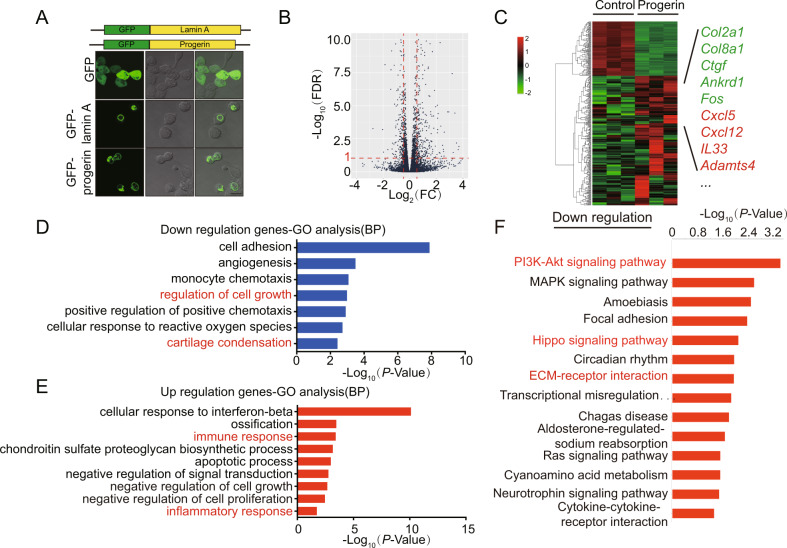
Progerin promotes inflammation and inhibits chondrocyte metabolism and proliferation. **A** The fusion protein of GFP with lamin A and progerin was constructed (Top), and immunofluorescence showed the nuclear architecture abnormalities changes after overexpression of the fusion protein in C3H10 cells (Bottom). **B** Volcano plot showing the number of differential genes between the progerin overexpression group and the control group. **C** Heatmap of RNA-sequencing data between progerin-overexpressing and control groups in C3H10 cells, *n* = 3 per group. Green represents downregulated genes, red represents upregulated genes. **D** GO analysis (Biological Pathway) of significantly downregulated (blue) genes in the progerin overexpression group versus the control group. **E** GO analysis (Biological Pathway) of significantly upregulated (red) genes in progerin overexpression group versus the control group. **F** Signal pathway analysis of genes significantly downregulated in progerin overexpression group compared with the control group.

We also know of patients with progeria, which is caused by progerin. Overexpression of progerin and deletion of *Zmpste24* both make the nuclear membrane abnormal. However, the relationship between the effects of both on chondrocytes is not well-understood. We examined the effects of overexpressed GFP-lamin A and GFP-progerin fusion plasmids in chondrocyte precursor cells and confirmed these results (Fig. 5A). Next, we overexpressed progerin in the C3H10 cell line and performed RNA-sequencing analysis. 106 genes were downregulated and 254 genes were upregulated in progerin-expressed cells with fold change greater than 1.5 and *P* value less than 0.05, compared with the control cells (Fig. 5B). Among the differential genes, *Col2a1*, *Ctgf* and other genes related to cartilage homeostasis maintenance and chondrocyte anabolism were downregulated, and *Cxcl5*, *Cxcl12*, *IL33*, and other inflammatory-related factors, *Adamts4* and other chondrocyte catabolism-related genes were upregulated (Fig. 5C). The biological functions of downregulated genes were mainly related to cell adhesion, angiogenesis, monocyte chemotaxis, regulation of cell growth and cartilage condensation. The biological functions of upregulated gene groups are mainly related to ossification, immune response, apoptotic process and inflammatory response (Fig. 5D, E). Signaling pathway analysis suggest that progerin accumulation changed the expression of different genes which are mainly related to the PI3K-Akt signaling pathway and Focal adhesion (Supplementary Fig. 3a). We next examined the pathways related to the downregulated genes and found that PI3K-Akt signaling pathway and Hippo signaling pathway was most significantly enriched in downregulated genes (Fig. 5F). We further analyzed all genes that were differential in the PI3K-Akt signaling pathway in the progerin group and found that *Col2a1*, a marker of chondrocyte anabolism, was significantly downregulated. In addition, genes such as *Sgk1*, and *Pdgfβ* in the PI3K-Akt signaling pathway were significantly downregulated (Supplementary Fig. 3b). These results indicated that *Zmpste24* deletion leads to osteoarthritis with dysregulated PI3K-Akt signaling and disordered chondrocyte metabolism. We also found that the downstream response molecules *Cyr61* and *Ctgf* in the Hippo signaling pathway were significantly inhibited, which indicated that the accumulation of progerin could also lead to the dysregulated Hippo signaling pathway (Supplementary Fig. 3c). These results are consistent with our previous findings that *Zmpste24* depletion results in slower cell proliferation and cellular senescence.

### ZMPSTE24 deficiency increases the level of H3K27me3 in chondrocyte senescence

**Fig. 6:**
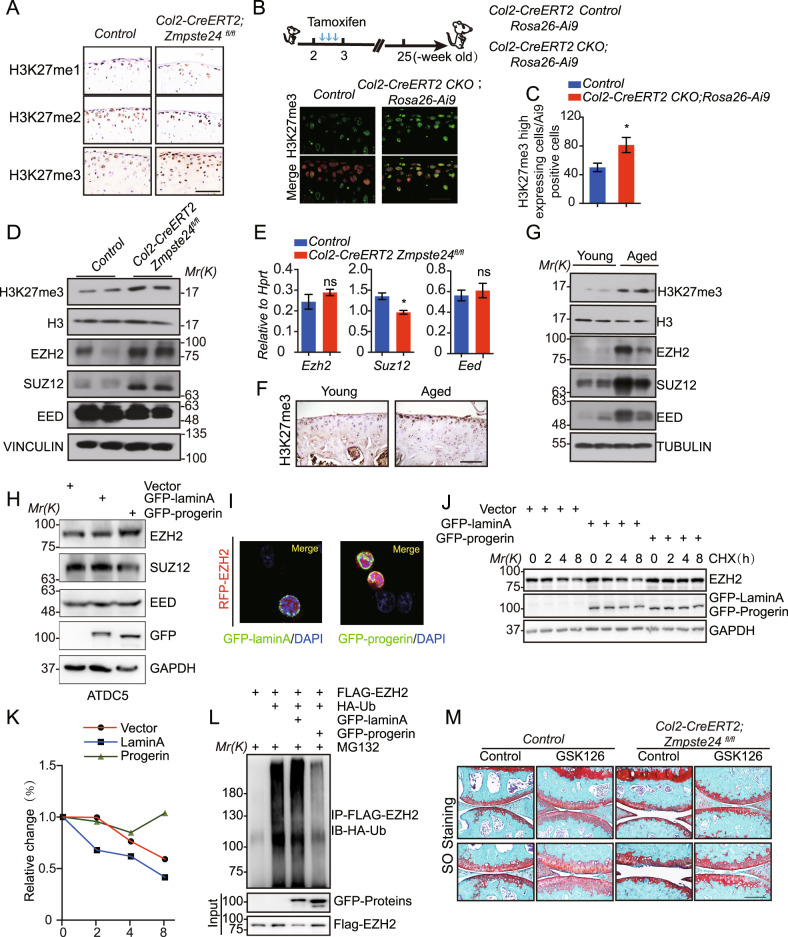
Increased the level of H3K27me3 in chondrocyte senescence. **A** Representative images of immunohistochemical staining of H3K27me1/2/3 in articular cartilage at 25-week-old. Scale bar = 50 μm. **B** Representative images of immunofluorescence staining of H3K27me3 (green) in articular cartilage at 25-week-old *Col2-CreERT2; Rosa26 Ai9; Zmpste24*^*fl/fl*^ (*Col2-CreERT2 CKO*); *Rosa26-Ai9* (*Tdtomato*) mice and *Control* mice. **C** Quantification of positive cells displayed by histogram (*n* = 3HPF, per group). Data represent the mean ± SD, *P < 0.05, with unpaired Student *t* test. **D** Western blot analysis of H3K27me3 and PRC2 complex members from articular cartilage of *Col2-CreERT2; Zmpste24*^*fl/fl*^ mice and *Control* littermates at 25-week-old, H3 and VINCULIN served as internal control. **E** RT-PCR analyses for relative RNA levels of the indicated genes in articular cartilage at 25-weeks-old. Expression levels of these genes were normalized and compared to control. Data represent the mean ± SD, *P < *0.05*, ns means no significance, with unpaired Student *t* test. **F** Representative images of immunohistochemical staining of H3K27me3 in sagittal sections of young and aged mice knee joints. Scale bar = 100 μm. **G** Western blot analysis of H3K27me3, EZH2, SUZ12, EED from cartilages of aged mice and young mice. H3 and TUBULIN served as internal control (*n* = 2, per group). **H** Western blot analysis of EZH2, EED, SUZ12 from ATDC5 cells infected with GFP-lamin-A and GFP-progerin lentivirus. VINCULIN as internal control. **I** Representative photos of confocal microscopy in HEK-293 cells expressing ectopic GFP-lamin A or progerin and RFP-EZH2 showing colocalization of GFP-lamin A/progerin and RFP-EZH2 on the nuclear lamina and in the nuclear interior (more than six sets of similar images were observed under the confocal microscope, and representative images were selected for presentation). **J** Representative western blots of GFP-lamin A or GFP-progerin lentivirus infection in CHX-treated ATDC5 cells. Protein levels of EZH2/GFP-lamin A/GFP-progerin were determined by western blot. GAPDH as the internal control. **K** Quantitative analysis of EZH2 bands in (**J**). **L** Ubiquitination analysis verified the potential of EZH2 protein ubiquitination modification. **M** Representative images of SO staining of hindlimbs from 25-week-old *Col2-CreERT2; Zmpste24*^*fl/fl*^ mice and Control littermates with injection GSK126 (*n* = 2–6 per group). Scale bar = 200 μm.

The epigenetic histone modification changes significantly during aging [32]. The methylation of Histone H3 lysine 27(H3K27) has been suggested as a key epigenetic regulation during aging process and there have been some controversial reports about the change of H3K27me3 in the aging process of different organs or different species [33–36]. How H3K27me3 changes during chondrocyte senescence has not been elucidated. We attempted to resolve these controversies using the animal models we constructed. Immunohistochemistry analysis demonstrated an upregulation of histone H3K27me3 modification in *Col2-CreERT2; Zmpste24*^*fl/fl*^ mice at 25-week-old after tamoxifen-induced (Fig. 6A). The upregulation of H3K27me3 were validated in *Zmpste24*-deleted cells which displayed Tdtomato signal in *Col2-CreERT2; ROSA Ai9* reporter mice (Fig. 6B, C). Western blot data also demonstrated the increase of total H3K27me3 level in articular cartilage from *Col2-CreERT2; Zmpste24*^*fl/fl*^ mice (Fig. 6D and Supplementary Fig. 4a).

Polycomb repressive complex 2 (PRC2), which contains four subunits, the catalytic subunit EZH2 or EZH1, and the scaffolding components SUZ12 and EED [37], is responsible of histone H3K27me3 methylation primarily at the promoters of target genes [38]. We next to assess whether changes in H3K27me3 level depend on the changes of the expression level of the subunits of PRC2 complex. Western blot analysis exhibited that EZH2 protein level was significantly increased in articular cartilage from *Col2-CreERT2; Zmpste24*^*fl/fl*^ mice compared with the control mice. In contrast, levels of *Ezh2* mRNA were hardly affected (Fig. 6E). These data suggest that deletion of *Zmpste24* results in upregulation of protein levels in the PRC2 complex, leading to aberrant H3K27me3 level. Interestingly, we also found the upregulation level of EZH2 expression with increased H3K27me3 level in 2-year-old natural-aging mice (Fig. 6F, G and Supplementary Fig. 4b), suggesting the changes in EZH2 lead to the increase in H3K27me3 during the development of osteoarthritis.

### Progerin interacts with EZH2 and increases the stability of EZH2

To further investigate the mechanism by which aging affect the H3K27me3 level. We hypothesize that nuclear structure abnormalities are the main factor. we stably expressed lamin A and progerin in ATDC5 cells and confirmed the increased EZH2 expression by progerin (Fig. 6H and Supplementary Fig. 4c). We next prompt to examine how progerin affect EZH2 protein level and found the colocalization of progerin and EZH2. However, it is barely to see the colocalization of lamin A and EZH2 (Fig. 6I). We next aimed to examine the effects of lamin A and progerin on the protein stability of EZH2. We expressed lamin A and progerin in ATDC5 cells. As shown in Fig. 6J, K, the expression of progerin could impair the degradation of endogenously expressed EZH2 which was determined by western blotting after CHX treatment for different time (Fig. 6J, K and Supplementary Fig. 4d). We also examine the ubiquitination of EZH2 and found that co-expression of progerin displayed lower EZH2 ubiquitination level (Fig. 6L and Supplementary Fig. 4e), suggesting that accumulated progerin could protect EZH2 from the degradation through the interference of EZH2 ubiquitination. Finally, we examined the effect of H3K27me3 inhibitor GSK126, which inhibits EZH2 catalytic activity, on the OA features of *Col2-CreERT2; Zmpste24*^*fl/fl*^ mice. As shown in Fig. 6F, GSK126 can partially alleviate osteoarthritis caused by *Zmpste24* deletion (Fig. 6M).

**Fig. 7:**
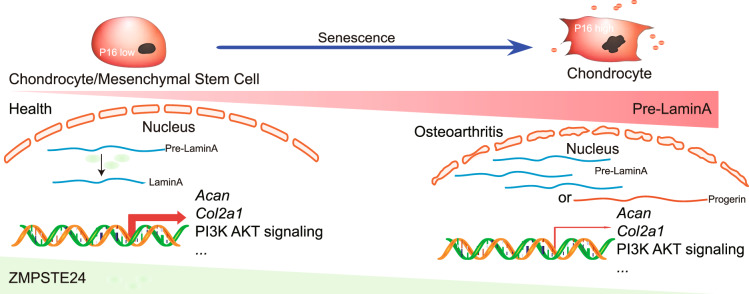
Schematic diagram showing the role of *Zmpste24* in osteoarthritis. Zmpste24 expression decreases with age, leading to the accumulation of Pre-LaminA in the cell nucleus. Deletion of *Zmpste24* in MSCs or chondrocytes leads to osteoarthritis in mice. Both models of *Zmpste24* deficiency and progerin overexpression in chondrocytes result in reduced chondrocyte anabolism and affect signalling pathways such as PI3K-AKT.

Overall, our results reveal that the reduction of *Zmpste24* in aging chondrocytes leads to nuclear architecture abnormal. The deletion of *Zmpste24* in MSCs and cartilage causes severe osteoarthritis with decreased cell proliferation and accelerated cell senescence. We also found that deletion of *Zmpste24* and overexpression of progerin lead to abnormalities in chondrocyte metabolism, slowed cell proliferation, and accelerated cellular aging (Fig. 7). Finally, we examined a long-time debate by using this animal model and found that chondrocyte senescence could promote H3K27me3 modification. Taken together, our study establishes a link between progeria and osteoarthritis and constructs an aging-induced osteoarthritis model, which would benefit the study of pathogenic mechanism of osteoarthritis and the discovery of a new therapeutic target for the treatment of osteoarthritis.

## Discussion

Our current research, constructed tissue-specific *Zmpste24* conditional knockout mice and demonstrated that *Zmpste24* deficiency in articular chondrocytes accelerated OA progression with increased inflammatory responses, decreased cell proliferation, and accelerated cellular senescence, confirming that cellular senescence is involved in aging-related OA development [8, 39, 40], and supporting that ZMPSTE24 plays a key role in the process of early aging by regulating the maturation of lamin A [22]. The mechanism study about ZMPSTE24 function in chondrocyte would benefit the understanding of the pathogenesis of OA.

There have been reports of a close connection between premature aging and natural aging [24]. In our database, we found that the accumulation of progerin accelerated the increase of inflammatory factors such as *Cxcl5* and *Cxcl12* chemotaxis, and the increase of these chemokines will induce an inflammatory response in cell populations, including macrophages, and accelerate the process of OA [41–43]. Our study fills in the conditions that induce the release of chemokines, such as *Cxcl5, Cxcl12*, and nuclear architecture abnormalities may be a switch. We also found that progerin promotes factors such as IL33, which promotes macrophage cells to trigger an inflammatory response, and our study suggests that progerin accumulation in chondrocytes will exacerbate malignant cell-to-cell communication. Blocking these inflammatory factors may have a certain alleviation effect on osteoarthritis caused by *Zmpste24* deficiency. Further work to elucidate how progerin induces the release of inflammatory factors will be worthy and the animal model constructed in this study would help to develop the molecular mechanism of inflammatory factor burst in aging OA.

In terms of mechanism, we found that two signaling pathways play an important role. Among them, the regulation of tissue development and cell proliferation by the Hippo signaling pathway is well known [44]. We established a link between *Zmpste24* and the Hippo signaling pathway. As a pathogenic gene of progeria, whether *Zmpste24* is also involved in the regulation of early development through the Hippo signaling pathway still needs to be studied. YAP1 has been shown to play an important role in chondrocyte development, fracture repair and osteoarthritis, and YAP1 is involved in the regulation of inflammatory factors [45, 46]. Although we injected mice with Tamoxifen for two weeks and observed no abnormalities in the growth plate of the mice, the possibility that the deletion of *Zmpste24* in earlier cell populations could affect chondrocyte development could not be ruled out. Another pathway we focus on is the PI3K-Akt signaling pathway. The function of this pathway in chondrocytes is relatively clear, but the association of *Zmpste24* with abnormal nuclear architecture abnormalities and calcium pathway is less studied [47]. Although we show that the change of nuclear architecture abnormalities will affect the PI3K-Akt signaling pathway, it is still worthy to investigate how nuclear architecture abnormalities affects the anabolic pathway.

In recent years, reports of histone modification and osteoarthritis have been increasing [48, 49]. Although the changes in H3K27me3 are well known in aging, but because of its differences in organization and species, there are many different opinions. However, there are increasing evidence that H3K27me3 may play an important role in OA, for example, Kdm6b as a demethylase of H3K27me3 has the function of protecting articular cartilage [48]. Metformin which has been previously characterized to activate AMPK, and AMPK suppresses EZH2-mediated Histone H3K27me3 [50]. In this study, we first used the *Zmpste24* knockout to successfully construct an OA model caused by articular cartilage aging. Although there have been reports of a close connection between premature aging and natural aging [24], our research at the mouse level shows that molecular mechanisms regulating H3K27me3 in natural-aging mice are similar to premature aging. And aging is a complex network, our study only focused on the methylation modification of histone H3 is limited. It is worthy to examine whether aging works in conjunction with other epigenetic modifications, for example, the dynamic balance between H3K36 trimethylation and H3K27me3 may also be closely related to OA caused by aging [51]. It will be meaningful work to use this model to develop other molecular mechanisms.

Overall, our studies first establish the *Zmpste24-*specific knockout mouse model in the skeletal system. We show that ZMPSTE24 knockdown in primary chondrocyte progenitor cells inhibits cell proliferation and accelerates cell senescence, *Zmpste24* deletion in articular chondrocytes leads to severe OA. Importantly, we established a link between HGPS and osteoarthritis and found that *Zmpste24* affects signaling pathways that are associated with physiological aging and other factors of this complex disease. *Zmpste24* will be one of the effective targets for the treatment of osteoarthritis, and the use of this animal model will accelerate the study of the pathogenic mechanism of aging osteoarthritis.

### Statistics

Statistical analysis was performed by unpaired, two-tailed Student’s *t* test for comparison between two groups using GraphPad Prism Software. A *P* value of less than 0.05 was considered statistically significant.

## Supplementary information

Supplementary figure legend

Supplymentary Figure 1

Supplymentary Figure 2

Supplymentary Figure 3

Supplymentary Figure 4

z24-OA-Cdd-aj-checklist

## Data Availability

All data generated or analyzed during this study are included in this published article (and its Supplementary information files). The datasets used and/or analyzed during the current study are available from the corresponding author on reasonable request.
