# Supplementary material for: Accelerated aging in articular cartilage by ZMPSTE24 deficiency leads to osteoarthritis with impaired metabolic signaling and epigenetic regulation
Source: Cell Death Dis. 2023 May 22;14(5):336. doi: 10.1038/s41419-023-05856-3 (PMC10203117; doi:10.1038/s41419-023-05856-3)
Supplement: Supplementary file 1 — Supplementary figure legend [file 41419_2023_5856_MOESM1_ESM.docx]

**Supplementary figure legend:**

**Supplementary Figure1.**

1. Gene expression of *Zmpste24* was detected after the primary chondrogenic progenitor cells were infected with *Gfp control* and *Zmpste24* lentivirus. The RNA levels normalized with HPRT were shown as fold change relative to control. Data represent the mean ± SD, ****P<0.001*, with One-way ANOVA followed by Turkey’s test.
2. The proliferation rates of primary chondrocyte progenitor cells infected with *Gfp control* and *Zmpste24* lentivirus were detected by cell proliferation assay. Absorbance was measured at 450 nm wavelength. n=5 per group.
3. Primary chondrocyte progenitor cells were induced to differentiate after infection with *Gfp control* and *Zmpste24* lentivirus and stained with alcian blue (n=3; three independent experiments). Scale bar=5mm.
4. Representative images of immunohistochemical staining of ZMPSTE24 in articular cartilage from *Zmpste24^-/-^* mice and *Control* littermates. Scale bar=100μm.

**Supplementary Figure2.**

1. Schematic representation of the Tamoxifen injection protocol and time points for collection of animal samples.
2. Immunofluorescence images show the distribution of Col2-positive cells after tamoxifen induction.

**Supplementary Figure3.**

1. Signal pathway analysis of genes significantly in progerin group compared with control group.
2. Gene expression of genes downstream of PI3K-Akt signaling pathway that varied significantly in RNA-sequencing results. Data represent the mean ± SD, **P<0.05*, ***P<0.01*, with unpaired student t-test.
3. Gene expression of genes related to cell proliferation downstream of Hippo signaling pathway that varied significantly in RNA-sequencing results. Data represent the mean ± SD,**P<0.05*, ***P<0.01*,with unpaired student t-test.

**Supplementary Figure4.**

1. Western blot corresponding to Figure 6D.
2. Western blot corresponding to Figure 6G.
3. Western blot corresponding to Figure 6H.
4. Western blot corresponding to Figure 6J.
5. Western blot corresponding to Figure 6L.
