# Supplementary figures and images for: Accelerated aging in articular cartilage by ZMPSTE24 deficiency leads to osteoarthritis with impaired metabolic signaling and epigenetic regulation
Source: Cell Death Dis. 2023 May 22;14(5):336. doi: 10.1038/s41419-023-05856-3 (PMC10203117; doi:10.1038/s41419-023-05856-3)

# Supplementary Figure 1

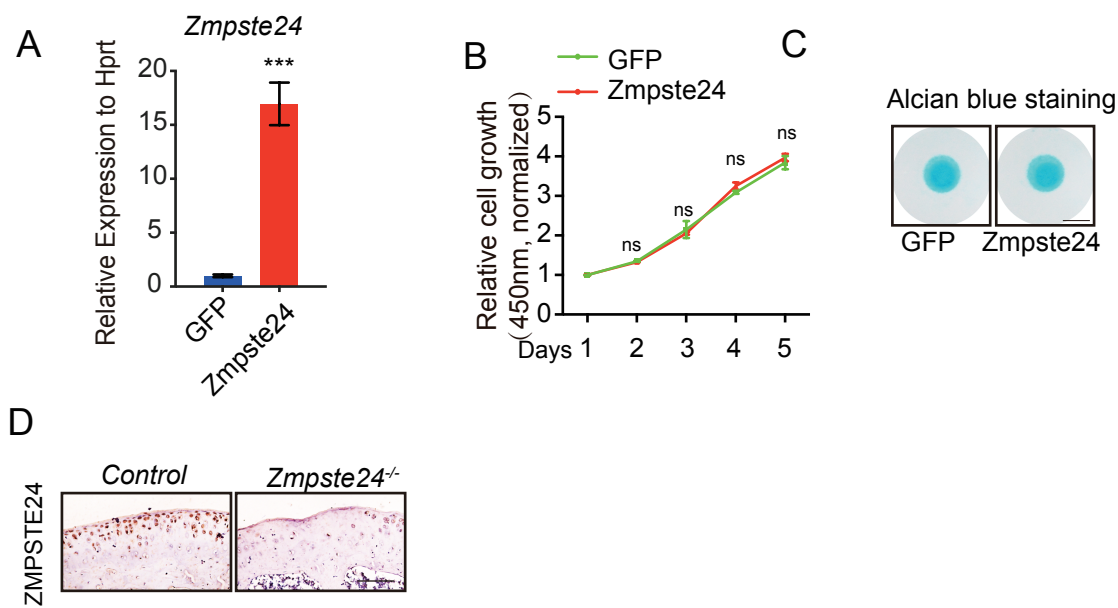

Supplement: Supplementary file 2 — Supplymentary Figure 1 [file 41419_2023_5856_MOESM2_ESM.pdf]

# Supplementary Figure 2

A

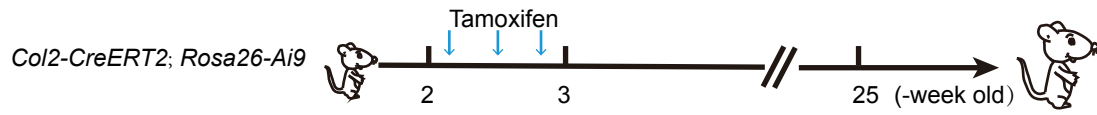

B

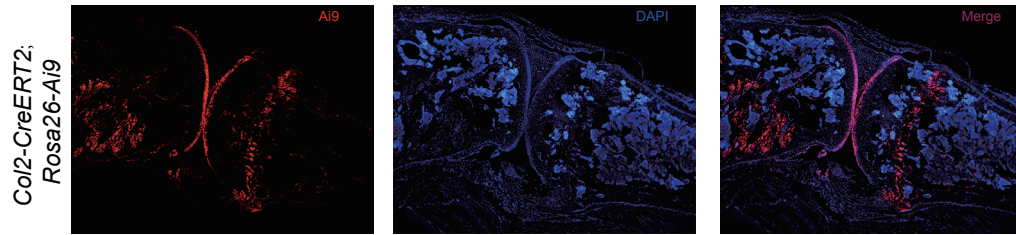

Supplement: Supplementary file 3 — Supplymentary Figure 2 [file 41419_2023_5856_MOESM3_ESM.pdf]

# Supplementary Figure 3

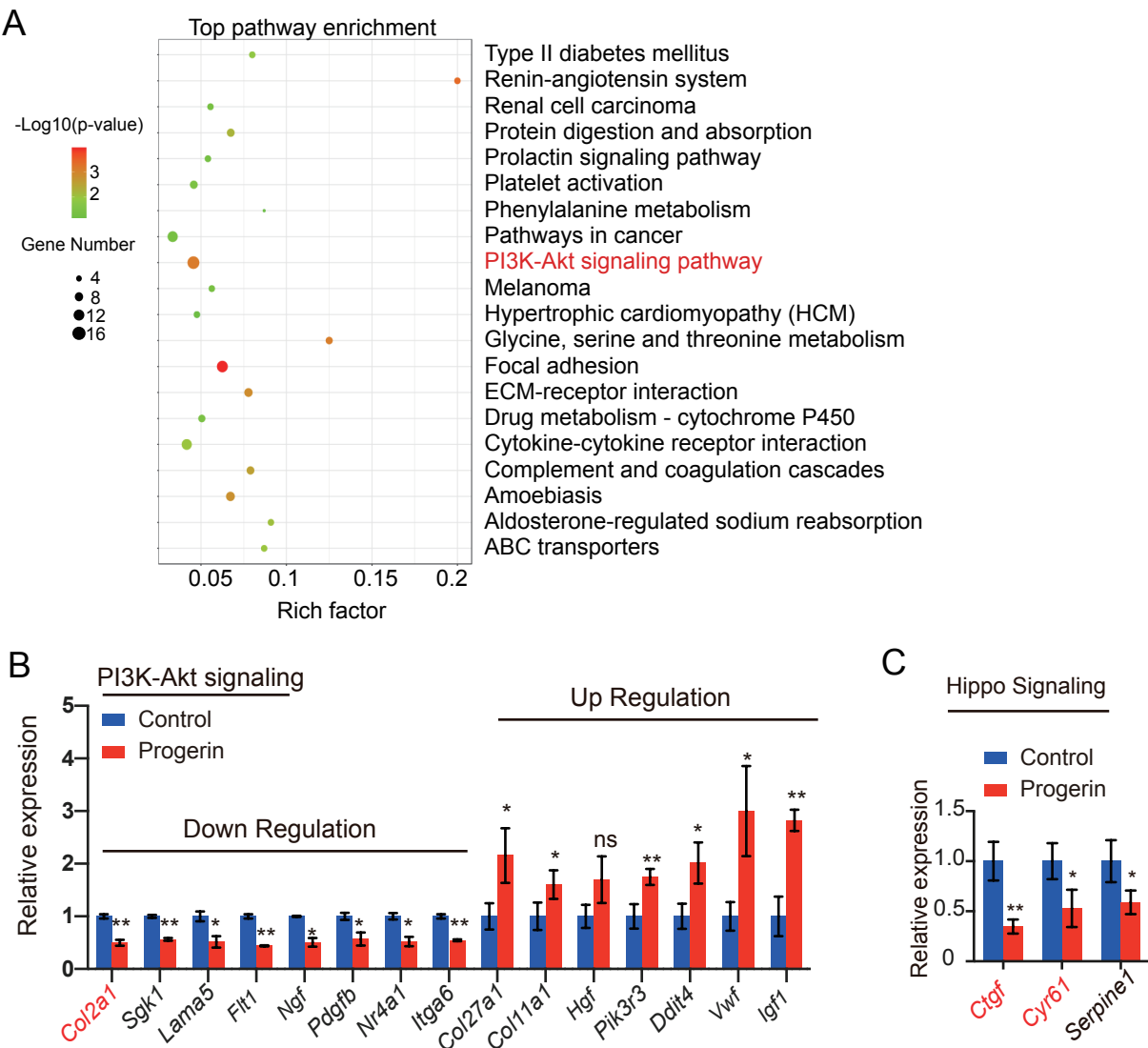

Supplement: Supplementary file 4 — Supplymentary Figure 3 [file 41419_2023_5856_MOESM4_ESM.pdf]

# Supplementary Figure 4

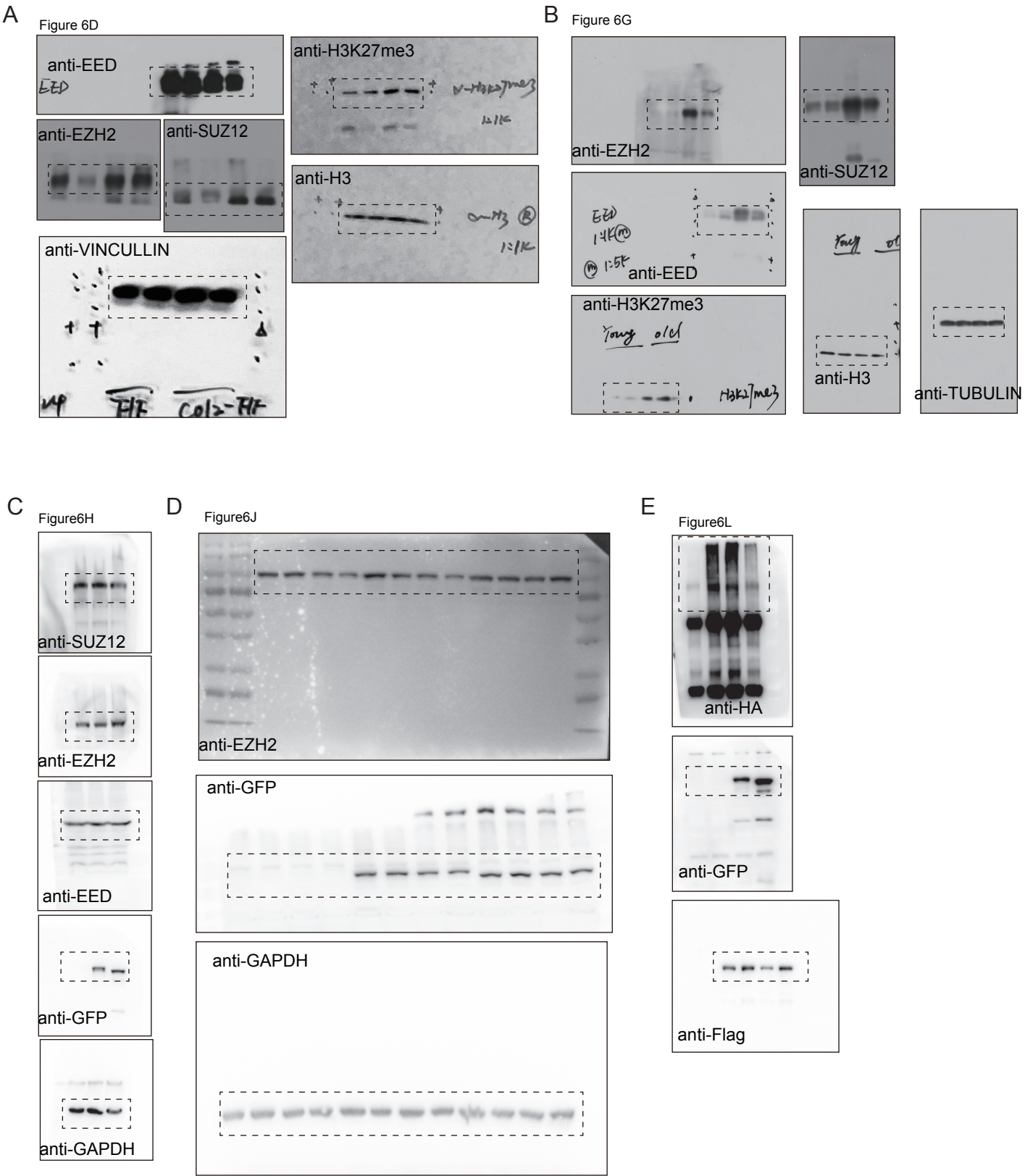

Supplement: Supplementary file 5 — Supplymentary Figure 4 [file 41419_2023_5856_MOESM5_ESM.pdf]
